# Glycemic indices of five varieties of dates in healthy and diabetic subjects

**DOI:** 10.1186/1475-2891-10-59

**Published:** 2011-05-28

**Authors:** Juma M Alkaabi, Bayan Al-Dabbagh, Shakeel Ahmad, Hussein F Saadi, Salah Gariballa, Mustafa Al Ghazali

**Affiliations:** 1 Department of Internal Medicine, Faculty of Medicine and Health Sciences, United Arab Emirates University, United Arab Emirates; 2 Abu Dhabi Food Control Authority, Abu Dhabi, United Arab Emirates

## Abstract

**Background:**

This study was designed to determine the glycemic indices of five commonly used varieties of dates in healthy subjects and their effects on postprandial glucose excursions in individuals with type 2 diabetes mellitus.

**Methods:**

Composition analysis was carried out for five types of dates (Tamer stage). The weights of the flesh of the dates equivalent to 50 g of available carbohydrates were calculated. The study subjects were thirteen healthy volunteers with a mean (± SD) age of 40.2 ± 6.7 years and ten participants with type 2 diabetes mellitus (controlled on lifestyle measures and/or metformin) with a mean HbA1c (± SD) of 6.6 ± (0.7%) and a mean age (± SD) of 40.8 ± 5.7 years. Each subject was tested on eight separate days with 50 g of glucose (on 3 occasions) and 50 g equivalent of available carbohydrates from the 5 varieties of date (each on one occasion). Capillary glucose was measured in the healthy subjects at 0, 15, 30, 45, 60, 90 and 120 min and for the diabetics at 0, 30, 60, 90, 120, 150 and 180 min. The glycemic indices were determined as ratios of the incremental areas under the response curves for the dates compared to glucose. Statistical analyses were performed using the Mann-Whitney U test and repeated measures analysis of variance.

**Results:**

Mean glycemic indices ± SEM of the dates for the healthy individuals were 54.0 ± 6.1, 53.5 ± 8.6, 46.3 ± 7.1, 49.1 ± 3.6 and 55.1 ± 7.7 for Fara'd, Lulu, Bo ma'an, Dabbas and Khalas, respectively. Corresponding values for those with type 2 diabetes were very similar (46.1 ± 6.2, 43.8 ± 7.7, 51.8 ± 6.9, 50.2 ± 3.9 and 53.0 ± 6.0). There were no statistically significant differences in the GIs between the control and the diabetic groups for the five types of dates, nor were there statistically significant differences among the dates' GIs (df = 4, F = 0.365, *p *= 0.83).

**Conclusion:**

The results show low glycemic indices for the five types of dates included in the study and that their consumption by diabetic individuals does not result in significant postprandial glucose excursions. These findings point to the potential benefits of dates for diabetic subjects when used in a healthy balanced diet.

**Trial Registration Number:**

ClinicalTrials.gov NCT01307904

## Background

The date palm (*Phoenix dactylifera *L.) is one of mankind's oldest cultivated plants. There are more than 2000 different varieties of dates [1], which have been used as food for over 6000 years. Dates are grown mostly between latitudes 10°N and 39°N [2] and are the most common fruit crop grown in the United Arab Emirates (UAE), occupying about 30% of the cultivated land [3].

Dates are rich in carbohydrates (total sugars, 44-88%), salts, minerals, vitamins, fatty acids (0.2-0.5%), proteins (2.3-5.6%), and fibers (6.4-11.5%) [4-6]. The development of the fruit is classified into four stages. Stage 1: 'Kimiri' stage, stage 2: 'Khalal' stage, stage 3: 'Rutab' stage and stage 4: 'Tamer' stage. The tamer stage is the final stage of maturation when the date has dried to a fairly firm consistency with a darker color [4].

The daily consumption of dates is a deeply rooted tradition in many societies, including those in the UAE. In Oman, which lies immediately adjacent to the UAE, the per capita daily consumption of dates is estimated at 55-164 grams and thus constitutes a vital component of the daily diet [7]. The UAE has developed rapidly over the last 40 years from a nomadic and trading economy into an emerging industrialized nation with a per-capita gross domestic product ranked seventh in the world (World Bank 2010). This economic growth has given rise to an abundance of food varieties and a decrease in physical activity which, in turn, has lead to a dramatic increase in the prevalence of obesity, metabolic syndrome, dyslipidemia, hypertension, prediabetes and diabetes [8-11].

The prevalence of diabetes mellitus (DM) in the UAE is currently the second highest in the world according to the International Diabetes Federation 2010 [8]. From a population-based study in the city of Al Ain in the UAE, the age-standardized rates for DM (diagnosed and undiagnosed) and pre-diabetes among 30-64 year olds were 29% and 24.2% respectively [9].

It is well documented that adherence to a healthy diet can improve glycemic control [12], may reduce glycosylated hemoglobin (HbA1c) levels [13-15], and when used in combination with other components of diabetes care, can further improve clinical and metabolic outcomes [13,14]. These observations have been translated into dietary guidelines for individuals with DM which include the recommendation that complex carbohydrates are preferable to simple carbohydrates [16,17].

The glycemic index (GI), first proposed in 1981 [18], is a system of classifying food items by glycemic response. The GI of a food depends upon the rapidity of digestion and absorption of its carbohydrates, which is determined largely by its physical and chemical properties. A particular food's GI is determined by measuring the rise in blood glucose after ingestion of a quantity of that food containing 50 g carbohydrate equivalent compared with the same amount of carbohydrate from a reference (such as glucose or white bread) taken by the same subject [19,20]. Using glucose as the reference, a GI of ≤ 55(i.e. ≤ 55% of the reference) is considered low, of 56-69 is considered medium, and of ≥ 70 is considered high [18].

In the UAE, the partaking in frequent snacks of dates (as often as four to five times per day) is a tradition. Our observation from clinical practice is that diabetic patients tend to receive conflicting messages from health educators regarding the advisability of consuming dates, with some suggesting restraint or even avoidance as a means to improve glycemic control. However, this appears to conflict with findings from previous studies demonstrating that dates have low to medium GIs [4,21-24]. We hypothesized that different types of commonly used dates would have low to medium GIs and therefore their consumption by diabetic subjects does not result in significant postprandial glucose excursions. The purpose of this study was therefore to evaluate the composition and the GIs of five common types of dates consumed in the UAE in both healthy and diabetic subjects. To the best of our knowledge, this is the first analysis of the composition and GIs of these particular dates among healthy and diabetic subjects. The results may help diabetic subjects and their health care providers in developing a diet that is both medically and culturally appropriate.

## Materials and methods

### Subjects

**Figure 1:**
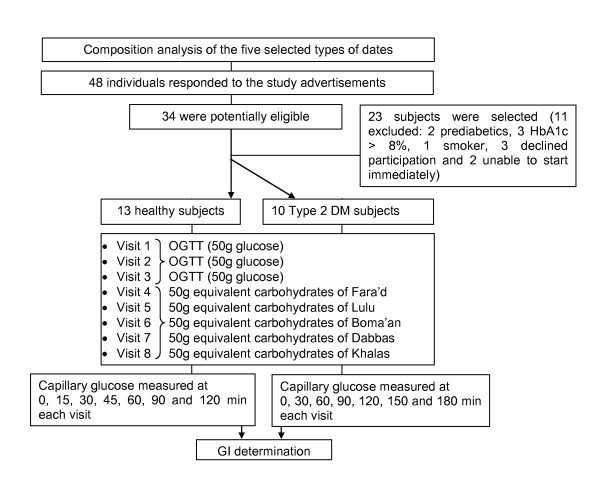
**Study flow chart**.

Study participants were recruited from local poster advertisements (Figure 1). After providing informed written consent, all volunteers completed an interviewer-administered questionnaire covering demographic data, tobacco and alcohol use, past medical and surgical history, co-morbidities, medications use and current health status. In patients known to have diabetes, information on disease onset, duration, and management was elicited. Each subject underwent a complete physical examination including measurements of blood pressure, pulse rate, height, weight, body mass index (BMI), body fat composition analysis using the Tanita TBF-410 Body Composition Analyzer (Tanita Corp., Tokyo, Japan) and measurement of waist circumference. Inclusion criteria required that those in the healthy group were indeed healthy and in the diabetes group that their diabetes was controlled (HbA1_c _≤ 8%) on diet with or without metformin. Exclusion criteria for both healthy and diabetic volunteers included morbid obesity (BMI > 40 kg/m^2^), pre diabetes, pregnancy, presence of gastroenterological disorders, alimentary tract surgery, a history of gastroenteritis in the prior six months, any alcohol intake, smoking, taking any medications (except metformin), poorly controlled diabetes (HbA1c > 8%) and the presence of chronic diseases (such as bronchial asthma or rheumatoid arthritis) or acute illness (such as upper respiratory tract or urinary tract infection).

Thirteen healthy subjects (7 females and 6 males) and ten volunteers with type 2 DM (5 females and 5 males) were enrolled for the study (Table 1). These numbers were chosen based on the literature where similar numbers had provided adequate power [19-22,25]. In order to reduce within and between-subject variability in GI measurements, subjects were asked to refrain from changing their eating and physical activity habits until the study was completed; i.e. subjects were advised to take their normal diet and to avoid unusual vigorous activity. In addition, we used capillary blood for the measurement of GIs instead of using venous samples and we also used oral glucose as a reference food three times in keeping with recommendations in the literature [26]. Patients on metformin (5 patients) were asked to take their usual dose of the drug before eating the test meal [19]. Prior to the GI studies, a fasting blood sample was obtained from all subjects for measurements utilizing a Beckman Coulter DXC800 (Beckman Instruments, Inc., Fullerton, CA, USA) auto-analyzer at the central laboratory of Tawam hospital, a tertiary referral hospital in Al Ain. These tests included a complete blood count, glucose and lipids, liver function tests, urea and electrolytes, urine protein and hemoglobin A1c.

**Table 1:** Demographic and other baseline characteristics of the studied subjects^1^

Parameter	Healthy (n = 13) Mean ± SD	Type 2 DM (n = 10) Mean ± SD
Age (Yrs)	40.2 ± 6.7	40.8 ± 5.7
Body weight (Kg)	75.4 ± 16.0	83 ± 16.7
Height (cm)	165.9 ± 8.0	163.9 ± 7.8
Body mass index (Kg/m^2^)	27.4 ± 4.1	30.7 ± 5.2
HbA1c (%)	5.8 ± 0.4	6.6 ± 0.7
Fasting blood glucose (mg/dl)	95.7 ± 6.5	116.1 ± 7.9
Waist circumference (cm)	Males: 97.3 ± 9.4 Females: 89.2 ± 12.6	Males: 101.3 ± 10.9 Females: 98.7 ± 9.2
Body fat composition (%)	Males: 29.8 ± 8.6 Females: 32.3 ± 8.2	Males: 30.0 ± 7.5 Females: 39.7 ± 6.1

^1 ^Data are presented as mean ± SD.

### Test foods

Fara'd, Lulu, Bo ma'an, Dabbas and Khalas, five varieties of date, very popular in the UAE, were chosen for this study. The same batches of packaged dates (Tamer stage) obtained from a local dates processing factory (Al Saad date factory, Al Ain, UAE) were used for all tests. The compositions of the dates (Tables 2, 3) were analyzed using standard methods (Association of Official Analytical Chemists, 2000) [27].

**Table 2:** Chemical compositions of the flesh of studied dates

Analysis	Fara'd	Lulu	Bo ma'an	Dabbas	Khalas
Moisture (%)	13.20	14.40	14.81	12.89	16.13
Crude fiber (%)	2.64	1.84	2.58	2.36	2.50
Fat (%)	0.12	0.15	0.06	0.15	0.12
Nitrogen (%)	0.186	0.240	0.224	0.259	0.191
Protein (%)	1.162	1.498	1.399	1.622	1.192
Fructose (%)	33.25	31.64	32.51	28.55	32.36
Glucose (%)	35.73	36.25	36.29	37.08	36.47
Sucrose (%)	0.91	1.07	0.16	2.26	BLD^2^
TRS^1 ^(%)	68.98	67.89	68.80	65.63	68.83

^1^TRS: total reducing sugars

^2^BLD: below limit of detection

**Table 3:** Trace elements and minerals content of the flesh of the studied dates^1^

Variety	Arsenic μg/kg	Cadmium μg/kg	Lead μg/kg	Calcium mg/kg	Sodium mg/kg	Iron mg/kg	Magnesium mg/kg	Phosphorus mg/kg	Manganese mg/kg	Zinc mg/kg
Fara'd	36.1	0.7	12.6	1170.5	192.1	2.256	1205.4	445.9	0.797	0.173
Lulu	29.2	2.2	45.5	517.7	93.4	7.94	411.1	338.3	1.268	1.415
Bo ma'an	68.8	0.9	24.7	288.6	47.7	7.369	561.0	543.8	2.341	1.172
Dabbas	36.4	0.9	25.3	846.5	91.1	3.495	604.9	411.8	4.293	0.820
Khalas	70.9	1.4	19.5	936.3	121.9	3.894	746.9	245.5	0.836	1.329

^1^Selenium < 0.14, Cobalt < 0.09 and Copper < 0.01 (mg/kg) for all five types of dates.

### Protocol

Glucose was measured in capillary blood samples using one of two One Touch II^® ^Lifescan glucometers (LifeScan, Inc., Milpitas, CA, USA), which were tested for accuracy and precision with the provided kits and against a Beckman Synchron CX7 laboratory analyzer (Beckman Instruments, Inc., Fullerton, CA, USA) which uses the glucose oxidase method. The coefficient of variation was 2.20-2.65% for both glucometers using three testing samples across the low, mid and high glucose ranges.

### Measurement of glycemic response

GI testing was carried out after an overnight fast on 8 occasions in every subject, each test being separated from the next by a "washout" day. As shown in Figure 1, the first 3 test days utilized 50 g of glucose dissolved in 250 ml water (Trutol^® ^50, Thermo Scientific) followed sequentially by 50g carbohydrate equivalents of the five selected dates (Table 4). The reference food (50 g of glucose) was tested on 3 alternating days in order to minimize day to day variation of glucose tolerance. Subjects were blinded to the type of the dates.

**Table 4:** Mean glycemic indices of dates in healthy and type 2 DM subjects^1^

Variety	Weight consumed (in g)*	Healthy subjects (n = 13) Mean GI ± SEM	Types 2 DM subjects (n = 10) Mean GI ± SEM
Fara'd	72.5	54.0 ± 6.1	46.1 ± 6.2
Lulu	73.6	53.5 ± 8.6	43.8 ± 7.7
Bo ma'an	72.7	46.3 ± 7.1	51.8 ± 6.9
Dabbas	76.2	49.1 ± 3.6	50.2 ± 3.9
Khalas	72.6	55.1 ± 7.7	53.0 ± 6.0

^1 ^Values are given as mean ± SEM.

*Weights of dates are equivalent to 50 g of available carbohydrate.

The dates were weighed using an H110 Sartor analytical scale (Sartorius AG, Goettingen, Germany), and consumed by all participants with 250 ml of water. Blood glucose was monitored during 2 hrs for the healthy individuals at 0, 15, 30, 45, 60, 90 and 120 min and over 3 hrs for the diabetics at 0, 30, 60, 90, 120, 150 and 180 min [19]. Areas under the curve (AUC) of blood glucose concentrations resulting from glucose given orally in a dose of 50 g with a corresponding oral carbohydrate load of 50 g were compared as previously described [19]. The 50 g of glucose was used as the reference food (GI = 100) against which all test dates were compared. The areas under the incremental glycemic-response curves for each date type were expressed as a percentage of the mean area under the three glucose curves for the same subject. The resulting values for all subjects were averaged to calculate the GI for each type of dates. The blood glucose levels used to calculate GIs were measured in our research laboratory in the Faculty of Medicine and Health Sciences, United Arab Emirates University between March and June 2010.

The study conformed to the requirements of the Declaration of Helsinki and was approved by Al Ain Medical District Human Research Ethics Committee (approval reference: 10/06) and was registered in a clinical trials registry (ClinicalTrials.gov Identifier:NCT01307904).

### Statistical analysis

Data were analyzed using Microsoft Excel 2003 and SPSS version 18 (SPSS Inc., Chicago, IL). For each dates meal the GI was measured by calculating the area under the curve using the formulae kindly provided by Professor Thomas Wolever from the University of Toronto-Canada. Standard descriptive statistics were used and results are presented as means and standard deviations (SD) or standard errors of the mean (SEM). In addition, for each type of date the GIs were compared between DM patients and controls using Mann-Whitney tests. Comparisons of GIs of the 5 different dates were carried out using repeated measures analysis of variance (ANOVA) on the combined (DM and controls) data set using groups as a between subjects factor. In all cases the statistical significance level was set at p ≤ 0.05.

## Results

The composition of the dates studied is shown in Table 2. The moisture content was 12-16%. The dates contained a high concentration of sugar, which is considered the main component (total reducing sugars, 65-69%). The highest concentration of available carbohydrate was in Fara'd dates and lowest in Dabbas dates. Crude fiber varied between 2-3%, fat 0.06 - 0.15% and protein 1.1-1.6% (Table 2). Thirteen salts and trace elements were also measured (Table 3).

There were 13 healthy subjects (7 females and 6 males) with a mean (± SD) age of 40.2 ± 6.7 years and BMI (± SD) of 27.4 ± 4.1 kg/m^2^, and 10 subjects with type 2 diabetes (F:M = 1:1) with a mean age (± SD) of 40.8 ± 5.7 years, BMI (±S D) of 30.7 ± 5.2 kg/m^2 ^and mean HbA1c (± SD) of 6.6 ± 0.7 (Table 3).

Table 4 shows the GI results for the five types of dates in healthy and diabetics subjects. The measured mean ± SEM GIs of the dates among healthy individuals were 54.0 ± 6.1, 53.5 ± 8.6, 46.3 ± 7.1, 49.1 ± 3.6 and 55.1 ± 7.7 for Fara'd, Lulu, Bo ma'an, Dabbas and Khalas dates, respectively. The mean ± SEM GIs among individuals with type 2 diabetes were very similar (46.1 ± 6.2, 43.8 ± 7.7, 51.8 ± 6.9, 50.2 ± 3.9 and 53.0 ± 6.0, respectively).

**Figure 2:**
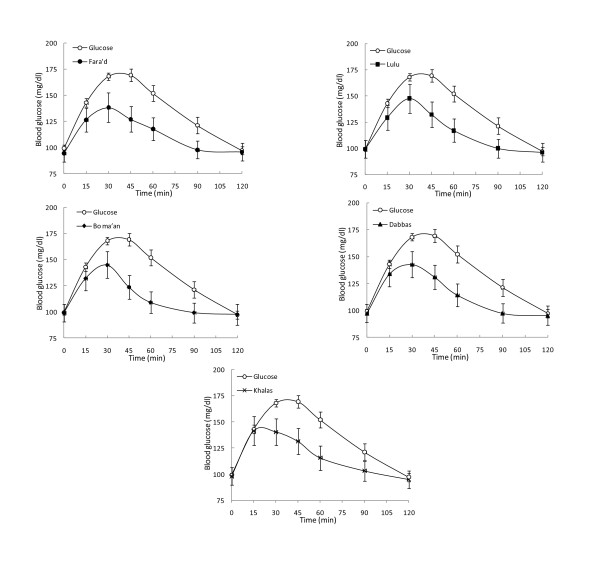
**Mean capillary glucose concentrations following ingestion of dates in healthy subjects**. Data are expressed as the changes in capillary glucose concentration from the fasting baseline concentration. Each data point represents the mean value for all the healthy subjects and the standard error of the mean.

**Figure 3:**
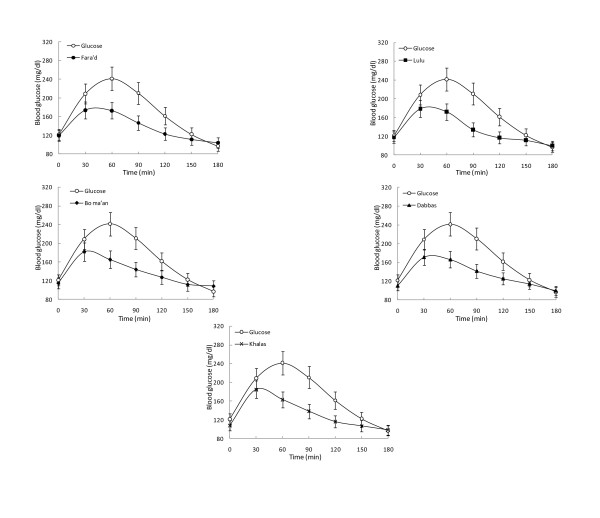
**Mean capillary glucose concentrations following ingestion of dates in subjects with type 2 diabetes**. Data are expressed as changes in capillary glucose concentration from the fasting baseline concentration. Each data point represents the mean value for all the healthy subjects and the standard error of the mean.

There were no statistically significant differences in the GIs between the DM and control groups (M-W tests), for any of the 5 dates, with p-values of 0.457, 0.495, 0.352, 0.951 and 0.901 for Farad, Lulu, Bo ma'an, Dabbas, and Khalas respectively. Nor was there significant heterogeneity in GIs among the 5 types of dates tested in GIs, ANOVA; (df = 4, F = 0.365, p = 0.83) within and between healthy and diabetic subjects. Given the absence of any evidence for heterogeneity in GIs among dates, no further post-hoc pairwise comparison were carried out. Figures 2 and 3 are graphic presentations of the GI changes in the healthy and diabetic subjects, respectively. The consumption of the five varieties of dates did not result in significant postprandial glucose excursions.

## Discussion

Dates are rich in certain nutrients and are widely consumed in many countries, particularly those within the Islamic world. They have been directly referred to in the Qur'an and in the Hadith (sayings of the Holy Prophet Mohammed).

Composition analysis of the pulp of the dates in our study was in keeping with the previously published literature [1,4,28]. The average water content of the dates we studied was 14.3%, consistent with the moisture content analysis (average 12.7%) of 13 varieties of dried dates [4]. Our studied types of dates are rich in carbohydrates with two monosaccharides, glucose and fructose, as the main reducing sugars. As fructose is twice as sweet as glucose, it plays an important role in the flavor and desirability of the dates. From a previously reported study carried out in the UAE, the total sugar content of 12 different varieties of dates varied from 44.3 to 64.1 g/100 g [29]. The carbohydrate content of dates depends on the type of date and the degree of ripeness with the highest concentration at the tamer stage.

The dietary fiber content varies depending on the type and degree of ripeness [4-6]. The percentage of dietary fiber decreases throughout the stages of maturation with the lowest percentage at the tamer stage [4,29]. Our measurement of the percentage of dietary fibers (2-3%) was similar to that previously reported [21,22]. The dietary fiber of dates at the tamer stage is mostly indigestible. The reported insoluble and soluble fiber components contribute 84-94% and 6-16% of total fiber respectively [28]. Higher ranges of total dietary fiber contents (7.2-14.9%) in 13 pre-packed date varieties from various countries have also been reported [30]. This variability in the reported dietary fiber content might be explained primarily by the different date varieties and the methods of measurement used. The consumption of 100g dates can provide 50 - 100% of the recommended daily amount of fiber [31].

The tested dates contain a higher percentage of protein than common fruits such as apples, oranges, bananas and grapes which contain 0.3%, 0.7%, 1.0% and 1.0% protein, respectively [32]. Our dates were also rich in salts, minerals and trace elements consistent with previous reports [4-6,28]. The trace elements are indispensable for proper functioning of a myriad of biochemical reactions, more particularly as enzyme cofactors in glucose metabolism, the organic derivatives yielded much better results than inorganic forms, likely because of better absorption [33].

The range of GIs of the five types of dates assessed in our study was 46 to 55 for healthy subjects and 43 to 53 for the type 2 diabetic patients. All of these date varieties, therefore, are low GI food items. In healthy subjects, the mean GIs reported by Miller et al for Khalas and Bo ma'an were 35.5 and 30.5 respectively [21] and the mean GI for Khalas with yoghurt mixed meal was 35.5 [22]. The GIs of three varieties of dates collected from various regions of Oman ranged between 47.6 and 57.7 [34]. Lock et al reported one date GI result of 61.6. However, that study was performed in pregnant women [23]. From international tables, the mean GI ± SEM for dates is 42 ± 4 [35]. In summary, the reported GI for dates classifies them as low to medium food items (mostly low GI food items). The low GI of dates can be attributed to their high fructose and dietary fiber content. In our study, the glucose: fructose ratio is approximately 1:1 consistent with previous publications [26,29,36]. A mean GI for fructose from 4 studies has been reported as 23 [37].

A diet low in GI may decrease the risk of coronary heart disease, gallbladder disease and breast cancer. Furthermore, a low GI diet demonstrably improves HbA1c levels, body weight and the lipid profile [38,15-43].

Whereas our estimates of the GIs among healthy volunteers are similar to previously reported values [21,22,34], we are not aware of similar studies on the effect of these dates on postprandial glucose excursions among diabetic subjects. Our results show that the consumption of the five varieties of dates did not result in significant postprandial glucose excursions, suggesting that such patients can consume dates in similar quantities to those used in this study without the risk of inducing undesirable postprandial excursions in blood glucose. The equivalent of 7-10 dates was used in each of our studies, which is similar to what is maximally eaten at a single sitting by UAE subjects. The caloric content, however, should be accounted for in any meal plan, as dates are rich in energy i.e. 100 g of flesh can provide approximately 314 kcal i.e. 11-15% of the total energy requirement per day for adults [36]. Further studies are needed to examine the GIs of dates at different stages of maturation and to measure the glycemic and metabolic profile responses to the consumption of dates in individuals with type 2 diabetics on different medication regimens, and in patients with type 1 diabetes.

## Limitations of the study

The test meals were not randomized and, ideally, the glucose meals should have been taken before, halfway and at the end of the test meals. We used only five varieties of dates (Tamer stage) hence the conclusions from this study may not be generalizable to all types of dates. The volunteers with type 2 DM were controlled on diet or metformin only. Thus, the results of this study cannot be extended to all patients with type 2 DM especially at advanced stages of the disease, i.e. those receiving multiple oral hypoglycemic agents or insulin.

## Conclusions

We have determined the composition of five common types of dates (Fara'd, Lulu, Bo ma'an, Dabbas and Khalas) and calculated their glycemic indices. Our results support the study hypothesis that the tested varieties of dates would have low GIs in healthy subjects and that their consumption by diabetic individuals does not result in significant postprandial glucose excursions. Future prospective studies are needed to evaluate the effects of long-term consumption of these dates on prevention of diabetes and other chronic diseases in the UAE, and on the control of hyperglycemia in subjects with diabetes.

## Abbreviations

GI: Glycemic Index; HbA1c: Glycated hemoglobin A1c; OGTT: Oral glucose tolerance test.

## Competing interests

The authors declare that they have no competing interests.

## Authors' contributions

AJ was the principal author, PI of the study, conceptualized and supervised the study, participated in the study design, analysis and interpretation of data and writing of the manuscript. BA participated in the study design and made substantial contributions to subjects' recruitment and performed laboratory and data analysis and co-wrote the paper. HS and SG participated in the design of the study and writing of the manuscript. SA and MG participated in the date's composition analysis. All authors read and approved the final manuscript.
